# Review: Cancer and neurodevelopmental disorders: multi-scale reasoning and computational guide

**DOI:** 10.3389/fcell.2024.1376639

**Published:** 2024-07-02

**Authors:** Ruth Nussinov, Bengi Ruken Yavuz, Habibe Cansu Demirel, M. Kaan Arici, Hyunbum Jang, Nurcan Tuncbag

**Affiliations:** ^1^ Computational Structural Biology Section, Frederick National Laboratory for Cancer Research in the Cancer Innovation Laboratory, National Cancer Institute, Frederick, MD, United States; ^2^ Department of Human Molecular Genetics and Biochemistry, Sackler School of Medicine, Tel Aviv University, Tel Aviv-Yafo, Israel; ^3^ Cancer Innovation Laboratory, National Cancer Institute, Frederick, MD, United States; ^4^ Graduate School of Sciences and Engineering, Koc University, Istanbul, Türkiye; ^5^ Graduate School of Informatics, Middle East Technical University, Ankara, Türkiye; ^6^ Department of Chemical and Biological Engineering, Koc University, Istanbul, Türkiye; ^7^ School of Medicine, Koc University, Istanbul, Türkiye; ^8^ Koc University Research Center for Translational Medicine (KUTTAM), Istanbul, Türkiye

**Keywords:** pediatric tumors, comorbidity, artificial intelligence, machine learning, mutations, transcriptomics, protein-protein interaction (PPI) networks, chromatin

## Abstract

The connection and causality between cancer and neurodevelopmental disorders have been puzzling. How can the same cellular pathways, proteins, and mutations lead to pathologies with vastly different clinical presentations? And why do individuals with neurodevelopmental disorders, such as autism and schizophrenia, face higher chances of cancer emerging throughout their lifetime? Our broad review emphasizes the multi-scale aspect of this type of reasoning. As these examples demonstrate, rather than focusing on a specific organ system or disease, we aim at the new understanding that can be gained. Within this framework, our review calls attention to computational strategies which can be powerful in discovering connections, causalities, predicting clinical outcomes, and are vital for drug discovery. Thus, rather than centering on the clinical features, we draw on the rapidly increasing data on the molecular level, including mutations, isoforms, three-dimensional structures, and expression levels of the respective disease-associated genes. Their integrated analysis, together with chromatin states, can delineate how, despite being connected, neurodevelopmental disorders and cancer differ, and how the same mutations can lead to different clinical symptoms. Here, we seek to uncover the emerging connection between cancer, including pediatric tumors, and neurodevelopmental disorders, and the tantalizing questions that this connection raises.

## 1 Introduction: background and premise

**FIGURE 1:**
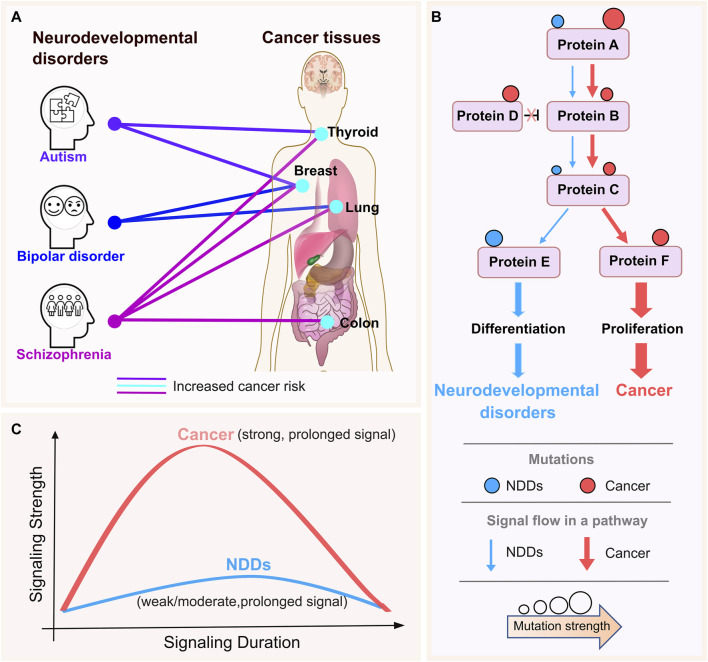
NDDs and cancer tissue connections with the premise. **(A)** The lines connect the neurodevelopmental disorders (NDDs) (autism, bipolar disorder, and schizophrenia) and cancer tissues that have been indicated to be associated with them in the literature. The colors are specific to NDDs for clarity. The thickness of the lines does not reflect any information. There is a small increase in the risk of thyroid and breast cancer in people with autism. Individuals diagnosed with schizophrenia have a roughly 50% higher chance of dying from cancer when compared to age- and sex-matched individuals in the general population, where there is a little increase in the risk of breast, oesophageal, and pancreatic cancer. There is a greater chance of developing colon, breast, lung and thyroid cancers among these individuals. In a similar vein, epidemiological research revealed a possible link between bipolar disorder and a higher risk of breast and lung cancers. **(B)** Schematic representation of signaling strength through a pathway for NDDs and cancer. NDDs and cancer recruits the same cellular pathways, proteins, and even the same mutations. Pink and blue circles represent cancer and NDD mutations, respectively. Circle size is depicting the strength of the activating mutation. Sizes of pink and blue arrows shows the signaling levels propagating to the downstream in cancer and NDDs, respectively. **(C)** Cancer mutations are strong, i.e., enabling strong and long signaling that propagates down the proliferation promoting pathways (solid, pink curve). In cancer, signaling is likely stronger than in wild type. In NDDs signal strength is weak/moderate and long fostering differentiation (solid, blue curve). Signal strength may also be affected by the rate of transcription initiation (Nussinov et al., 2023c) as observed in the repressed rate of nascent RNA transcription of highly methylated long genes in the brain through interaction of MeCP2 with the NCoR co-repressor complex which results in the devastating neurodevelopmental disorder Rett syndrome (Boxer et al., 2020).

The association between neurodevelopmental disorders (NDDs) and certain types of cancers has been implicated in several epidemiological studies. Compared to age- and sex-matched individuals in the general population, patients with schizophrenia have a roughly 50% higher risk of dying from cancer (Nordentoft et al., 2021), with seven quantitative studies of 1,162,971 participants yielding mortality risk from breast, colon, lung, and prostate cancer (Ni et al., 2019). Individuals with bipolar disorder and their unaffected siblings under 50 years of age had a higher chance of developing breast cancer than those in the control group. The correlation between bipolar disorder and the younger population’s susceptibility to bipolar disorder and higher cancer risk could indicate a genetic overlap in the pathophysiology of neurodevelopment (McGinty et al., 2012; Peng et al., 2021; Chen et al., 2022). When comorbid intellectual disability and/or birth defects are present, those with autism spectrum disorders are more likely than those without autism spectrum disorders to have cancer in their early years. After controlling for characteristics such as sex, birth year, parental age, etc., the link persisted and was not expected to be caused by other confounding variables (Liu et al., 2022). The relationships between bipolar disorder, autism, and schizophrenia and malignancies of the breast, colon, thyroid, and lung are summarized in Figure 1A; if a particular NDD has been linked to a greater cancer incidence in a tissue, we link them together with a line.

Epidemiological cohort studies opened the quest for the molecular mechanisms underlying NDDs and cancer - pathologies with distinct clinical presentations. Subsequent findings have suggested a cellular and organismal relationship between these pathologies. The Ras network impacts both diseases by aberrant regulation of the cell cycle (Nussinov et al., 2022b; Nussinov et al., 2023c). Two major mitogen-stimulated signaling pathways feed into the cell cycle (MacCorkle and Tan, 2005; Foijer and Te Riele, 2006; Sever and Brugge, 2015; Yang et al., 2017; Min et al., 2020). The first is MAPK, which controls cell division and is the major pathway in cell proliferation (Guo et al., 2020). The second is PI3K/AKT/PDK1/mTOR, the primary pathway in cell growth, thus differentiation (Saltzman, 2004; Li and Kirschner, 2014; Ruijtenberg and van den Heuvel, 2016). Proliferation and differentiation are vital to both cancer and NDDs. The fact that the same proteins in these pathways, and even the same mutated residues are involved (Marmion et al., 2023), raises compelling questions, including (i) why the phenotypic presentations are vastly different, and (ii) how we can develop a strategy to identify and predict NDD-related mutations and distinguish them from those related to cancer even though the same residue is mutated. If we are able to develop such a strategy, it may help in early NDD diagnosis. Epidemiologic surveys for multiple NDDs unearthed a clinically intriguing and abysmal connection: individuals afflicted with NDDs may face higher probabilities of developing cancer later in life (Nordentoft et al., 2021; Liu et al., 2022). We believe that addressing the link between these two pathologies will contribute to deciphering the underlying molecular mechanisms, treatment and preventive care.

Our premise is that the differences between cancer and NDDs are largely the outcome of the perturbation of signaling levels, which depend on (i) gene expression, which in turn, depends on cell type and state, and timing window (e.g., embryonic developmental stages or throughout life); (ii) homeostatic mechanisms that can block or enhance the signal; (iii) the strength of the activating mutation; (iv) the types and locations of additional mutations, and (v) the expression levels of specific isoforms of genes and regulators of proteins in the pathway. Expression levels indicate the role of chromatin structure. The sparseness of data makes single cell transcriptomics challenging, and structural networks may not be able to distinguish between isoforms whose sequences (and structures) are highly similar yet have different functions (Nussinov et al., 2022b; Nussinov et al., 2023c).

As to why the epidemiological connection, we reason that one possible clue is the established cancer statistics: A single mutation is insufficient to elicit cancer, although exactly how many mutations are required for cancer to emerge has been a debated question (Tomlinson et al., 2002; Tomasetti et al., 2015; Martincorena et al., 2017; Liu et al., 2023). Going back to the question we posed above, why then individuals with neurodevelopmental disorders, such as autism and schizophrenia, may face higher chances of cancer emerging throughout their lifetime? NDDs frequently involve germline mutations inherited from a parent who may not show the related phenotype. Activating mutations emerging during life can couple with the pre-existing germline mutations, strengthening the proliferative signaling and the likelihood of cancer. Such a scenario resembles latent driver mutations in cancer (Nussinov and Tsai, 2015; Nussinov et al., 2019a; Nussinov et al., 2023b). Latent drivers have low frequencies; thus, their translational potential may have escaped detection. However, when paired with another mutation in the same allele, they can drive cancer. Our comprehensive pan-cancer statistical analysis observed a significant occurrence of the double mutation pairs (Yavuz et al., 2023b). This scenario may manifest an otherwise unobserved NDD pathology in the parent. The computational challenge is to identify the impact of such mutations within phenotype (Nourbakhsh et al., 2024).

Here, we aim to enlist computations to discover how same pathway and same-gene mutations, and overexpression can preferentially lead to cancer and NDDs phenotypes, with the overarching goal of applying the learned knowledge to identify likely culpable genes/mutations (Figure 1B). Our hypothesis-driven proposal is consistent with early experimental indications (Marshall, 1995; Murphy et al., 2002; Murphy et al., 2004; Ben-Ari et al., 2010). Our premise is that under physiological conditions, mitogen-induced signaling that propagates down the pathway into the cell cycle is strong, but the bursts are of short duration (Figure 1C). In cancer, constitutive activating mutations and overexpression induce strong signaling that does not abate over time. A long duration of potent, too strong signaling can elicit oncogene induced senescence. In NDDs signal strength is moderate and long. Strong signaling bursts is expected to promote cell proliferation, weaker signaling to foster differentiation, and moderate/weak signal strength in aging may be associated with neurodegenerative diseases. Comparisons of NDDs and cancer—including the observed mutation types, transcriptomic data, protein-protein interaction (PPI) networks, and protein conformational dynamics (Jang et al., 2023b) —can suggest the (relative) signaling strength. We expect signaling strength in benign pediatric tumors (Li and Langhans, 2021), like cutaneous neurofibromas, to resemble that in NDDs. Individuals with NF1 mutations might be at risk of developing certain tumors earlier in life (Landry et al., 2021), with unidentified latent embryonic mutations rendering them genetically predisposed.

**FIGURE 2:**
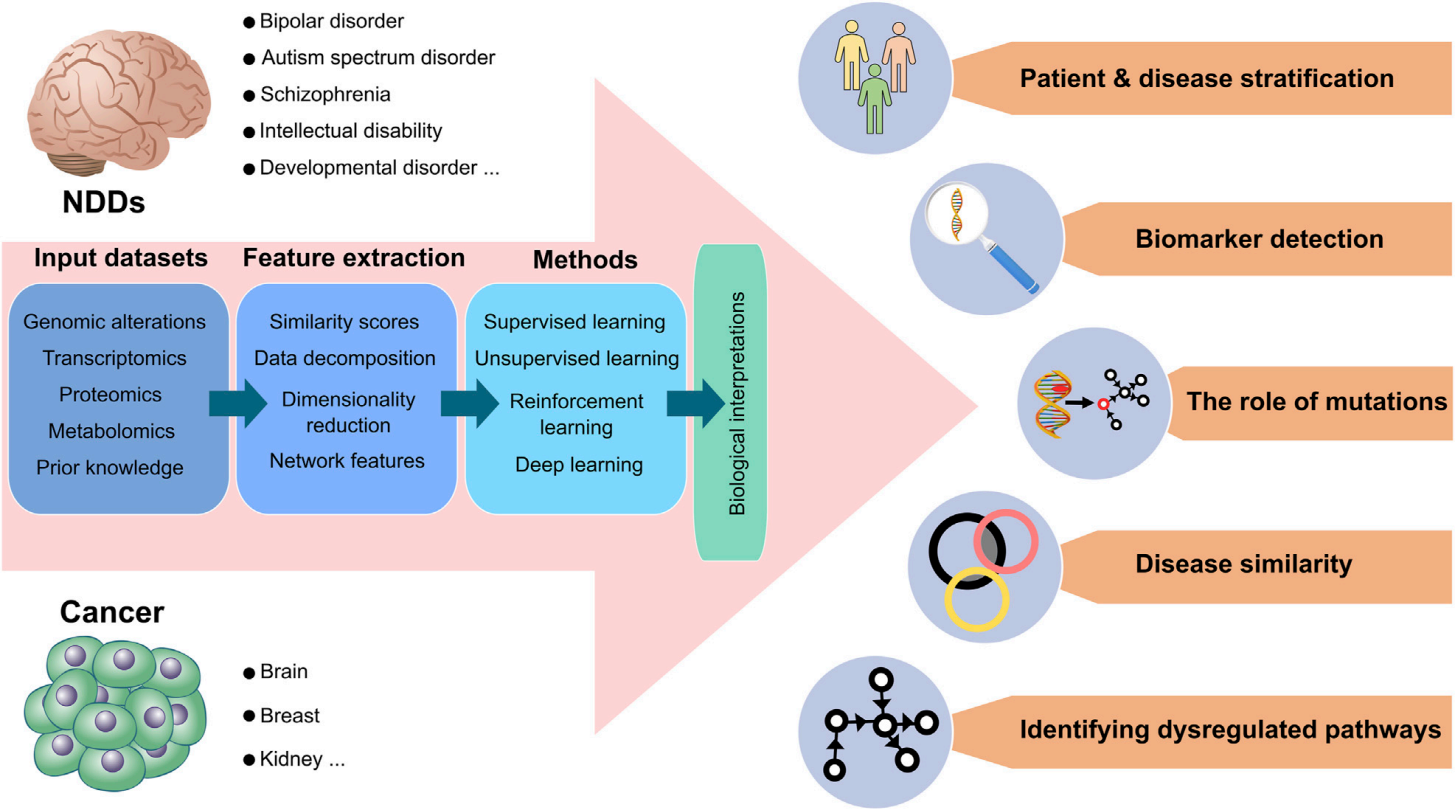
Data processing and potential applications for neurodevelopmental disorders and cancer. Current neurodevelopmental disorders (NDDs) data suggest that certain NDDs, including bipolar disorders (BDs), autism spectrum disorders (ASDs), and schizophrenia are more prone to develop certain cancer types, such as brain, breast, and kidney cancer. The sequential data process is demonstrated from left to right, respectively, for both cancer and NDDs. As input datasets, genomic alterations, transcriptomics, proteomics, metabolomics, and prior knowledge databases are mainly used in biological analysis. The input datasets are transformed into vectors by extracting significant feature sets through dimensionality reduction, similarity scores, data decomposition, and network-based methods. Computational methods can analyze extracted features by combining supervised, unsupervised, deep, and reinforcement learning methods. Eventually, all data processing allows various biological interpretations of high throughput inputs. Patients with NDDs and cancer are clustered and stratified based on molecular profiles so that different severities among patient groups can be categorized for precise medicine. Associated mutations, genomic variants, or critical genes can be predicted as biomarkers. Computational methods can determine how mutations affect the phenotypes of patients. Different subtypes of NDDs and cancer can be compared, considering their commonalities and discrepancies in significant feature sets. Detailed molecular alterations in signaling pathways can illuminate dysregulated pathways and their outcomes.

The significance of resolving the connection between cancer and NDDs (Nussinov et al., 2022b; c), coupled with advances in the computational sciences, machine capabilities, and the accumulation of experimental data across size scales suggests diverse approaches to decipher the connection and causality between these two complex processes. The challenge is in selecting the questions, the right tools (artificial intelligence (AI) based such as machine learning (ML), deep learning (DL), or other, advanced, or classical methods) to target them, and the protocols (Zeng et al., 2022). Among these, molecular dynamics simulations, which are essentially a single molecule approach, can also be considered as a predictive tool of cell phenotype (Nussinov et al., 2023b; a). Tool selection is also dependent on how much data (and of what quality) are available. Below, we provide an overview of the computational modeling and pipelines aiming to elucidate the connections and causalities. Figure 2 overviews the toolboxes and resources we envision for potential applications and data processing.

## 2 Computational modeling of the systems: network models

Cancer and NDDs catalyze and are impacted by dysfunctions in biological processes and pathways, including chromatin remodelers, PI3K/mTOR, and MAPK pathways (Zheng et al., 2018; Nussinov et al., 2023c). Therefore, the identification of functional items—modules or pathways—is a critical component in the comparison of the two diseases. Upon comprehensive characterization of the implicated pathways, it would also be possible to propose pertinent combination therapies for effective treatment (Nussinov et al., 2024). In such a task, network models are useful tools as they methodically address complex disorders by looking at dysregulated modules rather than the effects of individual mutations or genes in the conventional reductionist paradigm (Williams and Auwerx, 2015). Molecular networks exhibit high modularity, which can be defined as a high probability linkage among subsets of nodes. Specific modules frequently consist of different genes or proteins engaged in the same biological processes (Choobdar et al., 2019; Cui et al., 2019). Network models can also explain hypothetical causal mechanisms where multiple perturbations, such as mutations, are linked to pathways and processes (Nogales et al., 2022; Amgalan et al., 2023). In the mechanistic explanation of disease networks, cellular signal transduction is effectively depicted with causal knowledge such as kinase-transcription factor (TF) and TF-target interactions (Liu et al., 2019; Dugourd et al., 2021). Modular comparisons of network models can illuminate functional details of the commonalities and disparities between NDD and cancer.

## 3 Computational pipelines for elucidating the connections and causalities

Computational pipelines start with an input dataset, which may include genomic alterations, transcriptomics, proteomics, metabolomics, and any prior knowledge combinations (Milan Picard et al., 2021; Demirel et al., 2022) (Figure 2). Numerical features can be obtained from raw feature data using feature extraction (or selection) techniques. They can be used to eliminate redundant data so that the chosen learning algorithm only includes the most pertinent information in order to reach a biological interpretation. This would allow for inferences, such as the removal of diseased samples from healthy ones and the extraction of biomarkers for diagnostic purposes.

As the success of a computational pipeline heavily depends on the data, the selection of the appropriate dataset(s) relevant to the biological question and the necessary analyses is a crucial first step. Cancer-related datasets are available in many documented and controlled databases, such as TCGA (Cerami et al., 2012; Liu et al., 2018), AACR Project GENIE (Consortium, 2017), MSK-IMPACT Clinical Sequencing Cohort (Zehir et al., 2017), and primary and metastatic tumors from the Hartwig Medical Foundation (Martínez-Jiménez et al., 2023), thanks to the perpetual focus on cancers heretofore. While not being as widespread as cancer datasets, NDD-related datasets are also becoming increasingly available, as interest in understanding both NDDs and the cancer-NDD association has been on the rise (see Table 1).

**TABLE 1:** Some publicly available omics data sources for NDDs. Omics data sources for NDDs with the number of cases and controls are provided. Control type is also noted in the last column depending on the availability in the corresponding reference (otherwise specified with “-”).

Disease type	Study name	Data type(s)	Number of cases	Number of controls	References	Control type
Schizophrenia	SCHEMA	Mutation (WES, *de novo*)	24,248	97,322	Singh et al. (2022)	50,437 individuals without a known psychiatric diagnosis, the remaining not specified
Epilepsy	Epi25	Mutation (WES)	20,979	33,444	Epi et al. (2023)	Collected from multiple sources and not screened for neurological or neuropsychiatric conditions
Bipolar disorder	BipEx	Mutation (WES)	13,933	14,422	Palmer et al. (2022)	Without known psychiatric diagnosis
Autism spectrum disorder	ASC	Mutation (WES)	5,556	8,809	Buxbaum et al. (2012)	Ancestry-matchedcontrols
Autism spectrum disorder	ASC	*De novo* variants	6,430 probands	2,179	Buxbaum et al. (2012)	Unaffected siblings
Multitude of phenotypes	*de-novo* db	germline *de-novo* variants (WES/WGS)	23,098 trios	17,698	Turner et al. (2017)	-
Multitude of phenotypes	Deciphering Developmental Disorders (DDD)	exome sequencing and microarray analysis	1,133 trios	-	Wright et al. (2015)	-
Autism spectrum disorder	13 Orgo-Seq	bulk/single-cell RNA seq	13	12	Lim et al. (2022)	Without CNVs within the two ASD-associated loci in 16p11.2 and 15q11–13
Autism spectrum disorder	14 SFARI	WES, gene expression, metyhlation			Arpi and Simpson (2022)	-

The online single- and multi-omics data resources focused on cancer have been cataloged in Das et al.'s comprehensive review (Das et al., 2020). Several reviews covering emerging omics technologies for prognosis, early cancer screening, and diagnosis were published, along with a roadmap for multi-omics data integration techniques based on statistical methods and artificial intelligence (Biswas and Chakrabarti, 2020; Arjmand et al., 2022; Cai et al., 2022; Ma et al., 2022; He et al., 2023). Multi-omics integration techniques were applied to cancer research as well as to phosphoproteomics data (Mantini et al., 2021). Utilizing a proteogenomic approach to tumor investigation, the Clinical Proteomic Tumor Analysis Consortium (CPTAC) of the National Cancer Institute generates rich multi-omics datasets that link genomic anomalies to cancer descriptors (Li et al., 2023). Data on the full exome, whole genome, transcriptome, proteome, and phosphoproteome are provided for 10 cancer types including glioblastoma multiforme (GBM), lung adenocarcinoma (LUAD) and breast cancer (BRCA).

**FIGURE 3:**
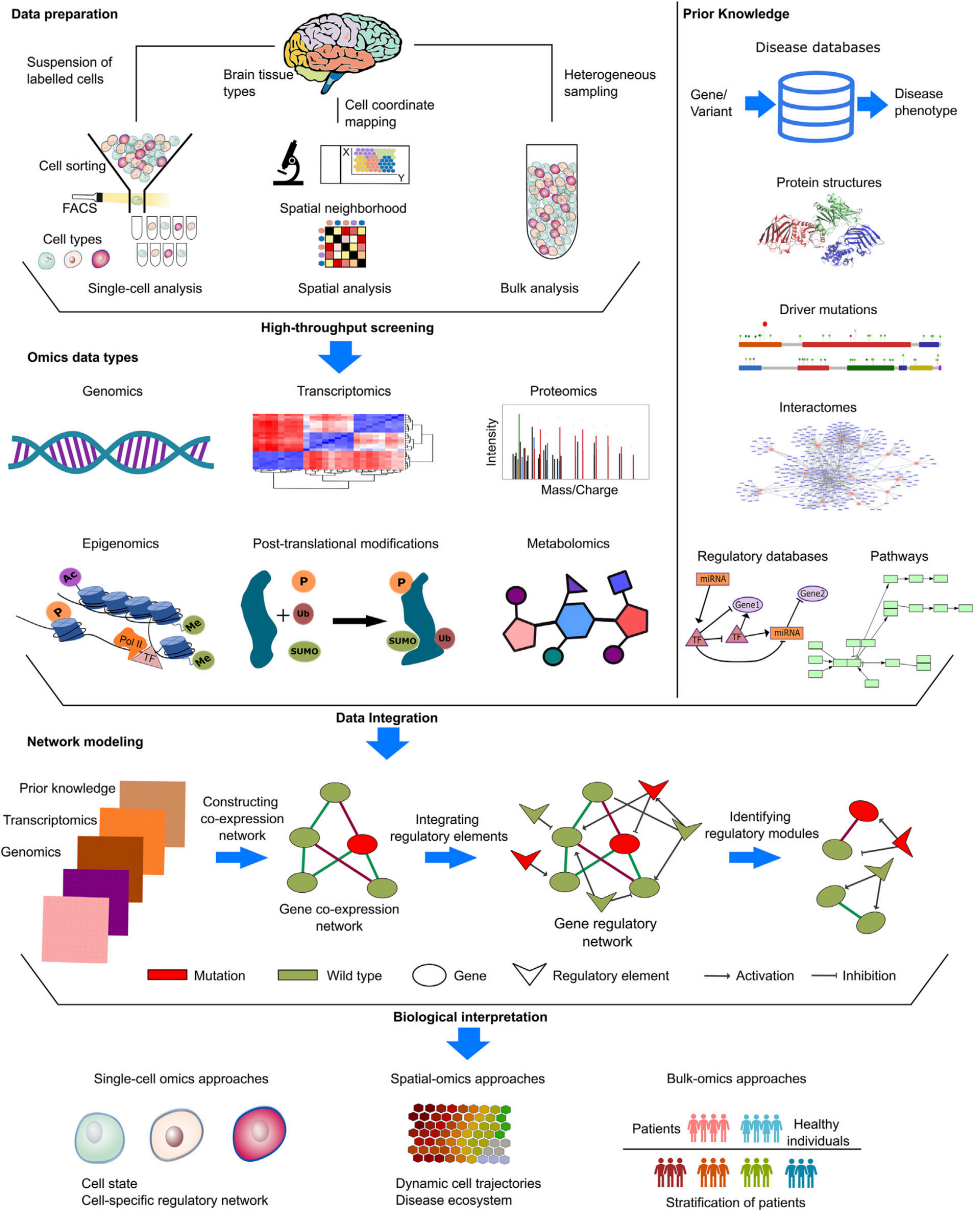
Input datasets in NDD and cancer. NDDs and cancer research provide various types of datasets, such as single-cell, spatial or bulk omics datasets. In single-cell methods, Fluorescence activated cell sorting (FACS) and Magnetic-activated cell sorting (MACS) are two common techniques used for the segregation of individual cells based on their distinct labeling and physical characteristics during the process of cell sorting. In spatial methods, samples are annoted with regional information by mapping the physical coordinates of cell and labelled for downstream analyses. In the context of bulk methods, the analysis entails the examination of heterogeneous samples originating from distinct microenvironments in a mixture form. These distinct methods provide complex insights from different molecular levels, covering genomics, transcriptomics, proteomics, metabolomics, and databases covering prior knowledge. Disease-specific mutations, copy number variations in patients, whole exome/genome sequencing data, and epigenetic alterations in patients are collected at the genomic level. Transcriptomics data are processed to identify significant expressions via differential expression in patients and comparative expression profiles in patient groups. Proteomics and metabolomics data, generated by mass spectrometry analysis, were assessed for phenotypic alterations at the translational level. Kinases are either active or inactive, depending on post-translational modifications. Thus, phosphoproteomics and other modification-specific proteomics gained prominence in NDDs and cancer research. In addition to sample-derived datasets, open-source databases integrate prior knowledge, just a few example of which are PDB (https://www.rcsb.org/) and AlphaFold DB (https://alphafold.ebi.ac.uk/) for protein structure, GRAND (https://grand.networkmedicine.org/) and TRRUST (https://www.grnpedia.org/trrust/) for regulatory networks, and STRING (https://string-db.org/) and HIPPIE (http://cbdm-01.zdv.uni-mainz.de/∼mschaefer/hippie/) for protein-protein interaction networks (interactome). Prior knowledge enables the identification and propagation of meaningful omic hits, allowing for the completion of unnoticed but associated information in cellular cascades. The integration of omics data with prior knowledge enables the establishment of causal links between different molecular levels. By examining the accessible chromatin areas, regulatory elements and their impact on transcriptomics can be assessed for biological interpretations in many areas including identification of cell states, cell specific regulatory networks, dynamic cell trajectories as well as patient stratification.

Computational studies mostly harness genomic and transcriptomic datasets to remedy biological questions (Figure 3). The genetic etiology of the NDDs is widely investigated through mutations (obtained with whole genome/exome or targeted sequencing) and copy number variations (CNVs). Comparisons of transcriptomic datasets, either bulk or single-cell type RNA-seq data, can identify disease. Further functional and molecular insight can be captured through proteomics and metabolomics approaches as well as post-translational modifications (Nascimento and Martins-de-Souza, 2015; Mueller and Meador-Woodruff, 2020; Murtaza et al., 2020; Ristori et al., 2020; Kopylov et al., 2023). Additionally, prior knowledge deposited in curated databases can provide information, including on protein structures (Berman et al., 2000; Varadi et al., 2021), interactomes (Alanis-Lobato et al., 2016), regulatory networks (Ben Guebila et al., 2021), metabolic pathways (Kanehisa et al., 2019), cancer drivers (Martinez-Jimenez et al., 2020), disease-related genes (Arpi and Simpson, 2022), and more, for sample-derived knowledge.

**FIGURE 4:**
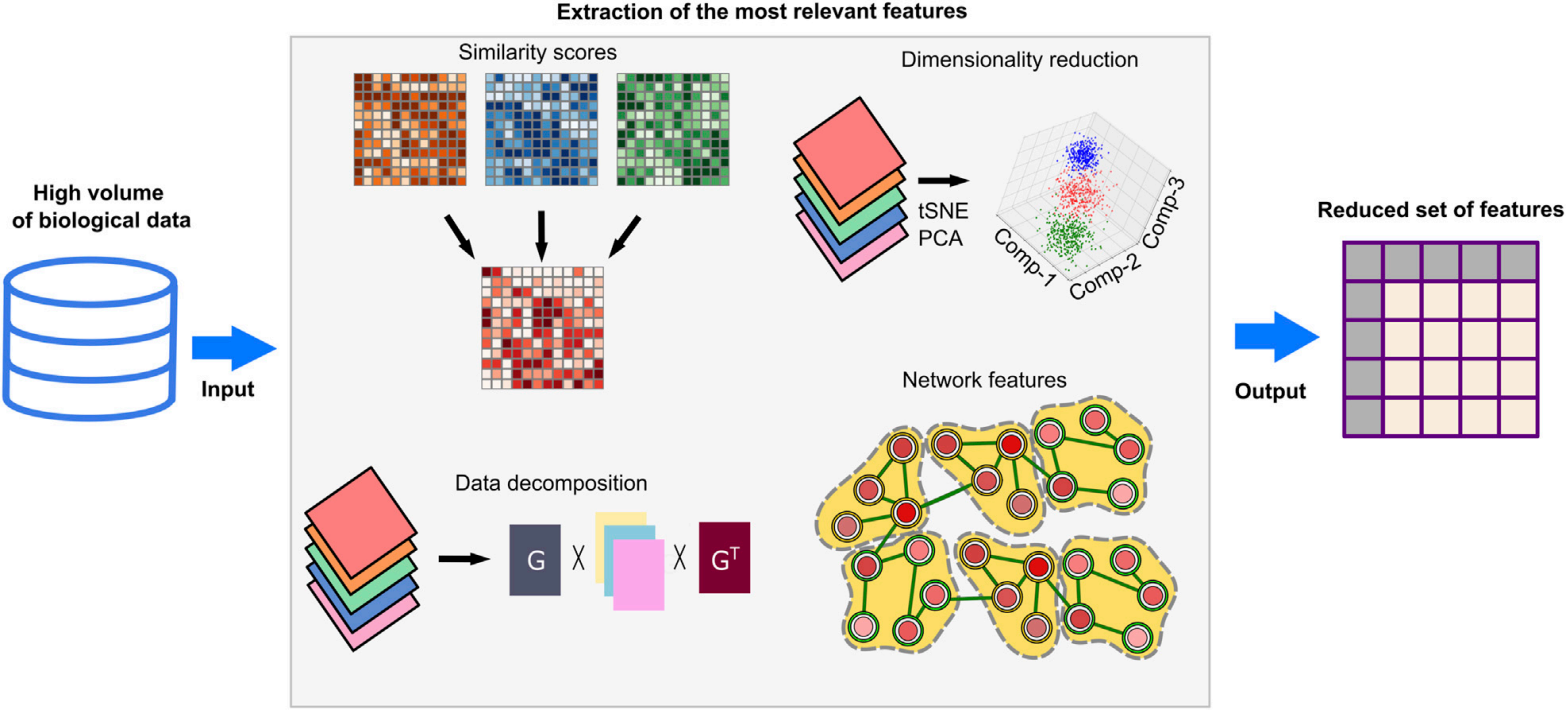
Feature extraction techniques. High-throughput omics datasets are noisy and need preprocessing to remove redundant features and extraneous before computational analyses and deducing biological interpretations. Feature extraction methods can simplify these datasets by eliminating irrelevant features, reducing high-dimensional structures, or dividing datasets into meaningful components. Similarity-based multi-omic data integrations identify the most relevant features at different molecular levels and generate lower dimensional data with minimal information loss. Dimensionality reduction methods such as PCA and tSNE can select the most explanatory variables or combine variables by generalizing complex datasets. By data decomposition techniques, input datasets are divided into vectorial compositions and recruited for downstream analysis. Network-based methods evaluate the topological features of context-specific networks and can detect biologically meaningful network modules and communities. As a result, associated biological knowledge including enriched pathways, biological processes or molecular functions can be inferred. The extracted biological knowledge in networks is transformed into vectors for downstream analyses.

Biological datasets are exceedingly complex and high dimensional as they cover thousands of genes, transcripts, and proteins. Feeding these datasets as-is into learning-based models will lead to incorporation of noisy or repetitive data and increase the computational costs. Feature extraction and selection methods prevent such problems as they enable extraction of the most relevant and simple features (Figure 4). Similarity matrices keep the pairwise similarities between the data points by utilizing measures such as Euclidean distance, cosine similarity, Jaccard similarity, and Pearson correlation coefficient (Guo et al., 2018). Dimensionality reduction methods–such as principal component analysis (PCA), t-distributed stochastic neighbor embedding (t-SNE), Uniform Manifold Approximation and Projection (UMAP), singular value decomposition (SVD), or non-negative matrix factorization (NMF)– can be applied to reduce the dimension of the similarity matrix along with the statistical tests and feature rankings to represent the data with minimum information loss (Meng et al., 2016; Guo et al., 2018; Reel et al., 2021). The above-mentioned dimensionality reduction methods like PCA, tSNE, and UMAP can be used for data visualization in 2D or 3D by reducing the dimensional complexity while keeping the maximum amount of information (Kim et al., 2018; Do and Canzar, 2021). Networks, such as regulatory networks, coexpression networks, or protein-protein interaction networks, transform multiple input datasets into more comprehensible data by using global and local features of networks (Li and Gao, 2019; Muzio et al., 2020). A community of interacting genes/proteins in these networks is mainly associated with specific biological knowledge. Thus, the use of communities in downstream methods can be advantageous to transform complex data into interpretable knowledge. Data decomposition approaches computationally break complex data problems into several components (Malod-Dognin et al., 2019). Associated and consistent components or detected -hidden but significant-components constitute feature sets.

The differences in the experimental protocols, tissue heterogeneity, and more, are expected to cause inherent noise among the datasets (Rahnenfuhrer et al., 2023). It is not uncommon to find inaccurate, or spurious correlations between unrelated data points due to the high-throughput nature of omics data, which typically result from a larger number of compared features (Clarke et al., 2008; Pudjihartono et al., 2022). As high-throughput data contains uninformative features, overfitting can lead to low generalization performance, and purging such features is inevitable. However, eliminating features without incorporating domain knowledge can lose critical information (Clarke et al., 2008). The aim of the study, the nature of data, the possible sources of noise should be considered carefully before using dimensionality reduction and other statistical methodologies. Although determining a single concrete map is challenging, the points discussed above could help reducing information loss and wrong derivations could be minimized.

**FIGURE 5:**
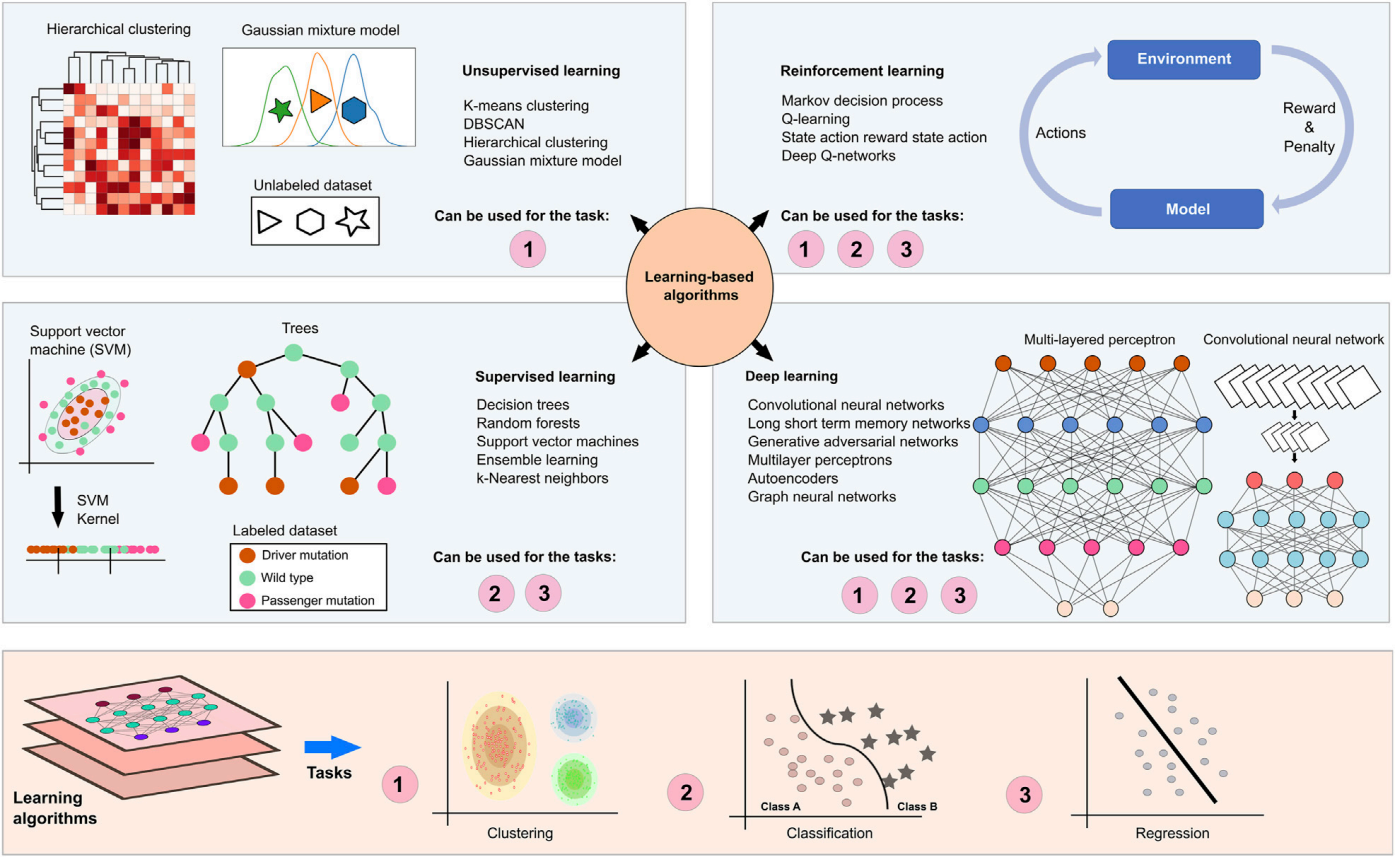
Learning based methods in NDDs and cancer research. Learning-based algorithms can facilitate the understanding of biological phenomena such as the effect of a mutation, molecular stratification of patients, and similarities/differences among complex diseases. Unlabeled sampling pools are recruited in unsupervised learning algorithms, such as k-means clustering, hierarchical clustering and Gaussian mixture models for clustering samples. On the other hand, patients and samples in the known phenotypic spectrum can be labeled based on prior knowledge, which allows recruiting supervised learning algorithms such as decision trees, random forests, support vector machines, ensemble learning, k-nearest neighbors for classification and regression analysis. Deep learning algorithms provide a substantial computational power to examine high dimensional datasets through the as a consequence of their multi-layered architecture. A multilayered perceptron utilizes feedforward activation functions to propagate information to the next layers and backpropagation to iteratively adjust the weights in hidden layers that are placed between input and output layers. These functions optimize inputs and weights in layers to generate outputs. In the layers of a convolutional neural network, convolution and pooling functions transform data matrices into smaller matrices, constructing feature maps. Fully connected feedforward neural networks learn and classify pooled features. Also, reinforcement learning algorithms utilize real-life scenarios by holding dynamic patient regimens and personalized medicines. The algorithm receives either rewards or penalties for its actions and goals to maximize the total reward.

Learning-based algorithms try to learn from the data to be able to predict the correct or relatable outcomes (Figure 5). Supervised learning techniques such as regression analysis, random forest, and decision trees require labeled samples, such as diseased *versus* control, where the algorithm can create a logical connection between the labels and the data to label possible unknown samples. Unsupervised learning methods such as clustering algorithms and Gaussian mixture models, on the other hand, do not depend on such labels and try to come up with common patterns based on the data itself rather than the labels. Reinforcement learning algorithms aim to maximize long-term rewards by learning from the rewards and penalties based on their actions. Such algorithms can be useful for patient-specific treatment regimens where selections are based on the biological characteristics and treatment response of the patients (Eckardt et al., 2021). DL is based on artificial neural networks and utilizes a multilayered learning structure that may include supervised, unsupervised, and semi-supervised methods to extract non-linear and complex features from high-dimensional datasets. DL models can be used for various biological problems from disease assessment to subtyping and survival analysis to precision medicine (Tran et al., 2021).

## 4 AI, ML, DL, and other computational methods in tackling NDDs

Numerous computational techniques have been used to study cancer, taking advantage of massive and quickly expanding databases. In addition to medical records, patient populations, cancer types, and clinical presentations, there are large databases of sequences, mutations, transcriptomics, and experimental 3D structures. Over the last years, computations related to NDDs have also been ramped up (Qi et al., 2016; Jiang et al., 2022). However, to date, they are of a different nature. They include a computational perspective on autism (Rosenberg et al., 2015), including exploiting ML models to understand and diagnose its pathogenesis in the context of complex neurodevelopmental heterogeneity (Jacob et al., 2019). They also include computational neuroscience approaches to identify precise, objective, and quantifiable markers of autism spectrum disorders (ASD) in physiological, behavioral, and neural processing (Computational Models of Autism) and computational analysis of neurodevelopmental phenotypes to harmonize clinical features (Lewis-Smith et al., 2022). These NDDs-related computations are associated with features at a different size scale than the molecular-level methods discussed here. Only one literature record compared the gene expression profiles of ASD frontal cortex tissues and 22 cancer types obtained by differential expression meta-analysis with reported gene, pathway, and drug set-based overlaps (Forés-Martos et al., 2019). The results suggested that brain, kidney, thyroid, and pancreatic cancers are candidates for direct comorbid associations with ASD, whereas lung and prostate cancers are candidates for inverse comorbid associations with ASD.

The most common NDDs are ASD and attention-deficit/hyperactivity disorder (ADHD). Other common NDDs include cerebral palsy, communication disorders, intellectual disabilities, learning disorders, and neurodevelopmental motor disorders (FamilieSCN2A Foundation, 2023). Intellectual disorder (ID), ASD, ADHD, schizophrenia, and bipolar disorder (BD) display a neurodevelopmental continuum that can be explained by gene nucleotide substitutions and copy number variants, e.g., nonsense mutations or splice variants. For example, in ASD, there is a strong association between mutations in *CDH8* (a gene encoding Cadherin 8, diseases associated with which include craniofacial-deafness-hand syndrome and ectodermal dysplasia) (Jiang et al., 2022). Similarly, 16p11.2 with duplication resulting in a low weight; a small head size (microcephaly), and with deletions playing a major role in developmental delay, especially in speech and language (Rosenfeld et al., 2010). Affected individuals also have an increased risk of behavioral problems, and *SCN2A* is commonly associated with early-onset epilepsy and linked to ASD and developmental delay (Morris-Rosendahl and Crocq, 2020; FamilieSCN2A Foundation, 2023).

25 distinct classes of brain cells originating from the primary motor cortex of the mammalians, mouse, marmoset and human, were identified, including 16 different neuronal classes, each composed of multiple subtypes (Network, 2021). The locations of cells related to the NDDs differ, e.g., cerebral palsy-related cells are in the part of the brain that controls movement, likely differing from that of ID. At the same time, comorbidity between certain NDD types suggests some adjoining genes partaking in a common chromosomal deletion or CNVs as in ASD 16p11.2 deletion. The mutations might be present on genes depending on the specific cell types and states, and these genes may exhibit varying levels of co-expression throughout embryonic brain development. Comorbidity between the NDDs is frequent. 22%–83% of children with ASD have symptoms that satisfy ADHD, and 30%–65% of children with ADHD have ASD symptoms (Morris-Rosendahl and Crocq, 2020). Studies focusing on mental disorders in children aged 7 through 12 have also observed that 55% of the children were diagnosed with at least one NDD. 40% did not have a diagnosed comorbid condition, and 26% had an anxiety disorder (Morris-Rosendahl and Crocq, 2020). ID, ASD, ADHD, and schizophrenia share specific genetic risks (Owen et al., 2011). Data suggests that in addition, intellectual disabilities share phenotypes with schizophrenia (Singh et al., 2017), further supporting the NDDs continuum (Owen and O'Donovan, 2017).

Below, we first describe computational studies in NDDs. While we do not dedicate a separate section to cancer-related computational studies, we encourage interested readers to explore recent reviews that focus on cancer and machine learning (Zhang et al., 2023; Ma et al., 2024; Unger and Kather, 2024) in addition to the ones mentioned earlier. We then proceed to the connection between NDDs and cancer. Finally, we provide some computational pipelines for such explorations.

### 4.1 Disease *versus* healthy sample classification

Most of the publicly available genomic datasets for NDDs were derived from case and control studies. As a result, for each type of genomic data, there are both diseased and physiological samples. By using supervised techniques on labeled input and outputs in datasets, it may be possible to classify disease *versus* healthy cases. Unsupervised clustering methods can successfully distinguish between the two states without labeled information.

Disease diagnosis is one of the most common goals of ML applications in precision medicine. The ability to separate healthy and diseased samples based on given data opens the door to early treatment options as well as detection of diseases that are difficult to identify. Approaches have been proposed for the detection of NDDs with varying performance metrics. Among the ways of assessing the performance measure of an AI algorithm, accuracy is one of the most common, indicating the proportion of cases that are correctly predicted (Erickson, 2021). Trakadis et al. used gradient-boosted trees with regularization for rare variants obtained from whole exome sequencing data to predict individuals at high risk for schizophrenia. They obtained an 85.7% accuracy (Trakadis et al., 2019). Liu et al. also focused on mutations for distinguishing ADHD cases from controls. They computed *p*-values for the association of single nucleotide polymorphisms (SNPs) with ADHD traits and selected SNP subsets based on different *p*-value thresholds. Their convolutional neural network model achieved an accuracy higher than 90% for the dataset with the most stringent *p*-value cutoff (Liu et al., 2021). Li et al. developed a deep canonically correlated sparse autoencoder model and tested it using SNP and functional magnetic resonance imaging (fMRI) data (Li et al., 2020). For schizophrenia classification, the accuracy with the SNP data was much higher (∼95%) compared to fMRI data (∼85%).

Some approaches highly prioritize decreasing the false positive rates even if it also causes a decrease in the number of total predictions or the true positive rate. They ensure that no control cases are falsely predicted as disease cases since an incorrect diagnosis of a healthy person may lead to severe outcomes. For example, ODIN (Oracle for DIsorder prediction) utilized *de novo* likely gene disruptive variants and gene similarity scores obtained from brain co-expression data to predict ASD and ID cases (Huynh and Hormozdiari, 2018). Although the method could only predict a subset of patients, it delivered a very low false positive rate and could also detect a group of samples that do not have any mutations on known frequently mutated genes. Similarly, Chow and Hormozdiari used shallow neural networks with ASD, ID, and developmental disorder *de novo* variants by also incorporating measures of gene constraint and conservation information (Chow and Hormozdiari, 2023). Their method could capture more than 30% of the cases with a false positive rate smaller than 1%.

Instead of focusing only on genetic data such as the presence of variants or gene expression, some studies also add data types such as fMRI imaging to obtain better performance. For instance, both Yang et al. and Lin et al. endeavored to classify a cohort of 40 individuals, consisting of 20 healthy subjects and 20 schizophrenia patients, by integrating SNP data with fMRI data (Yang et al., 2010; Lin et al., 2011). They assessed the efficacy of using each dataset independently and evaluated their performance by enlisting 39 participants for training and one for testing. The integration of SNP-fMRI exhibited superior performance in both studies. Yang et al. obtained an accuracy of 87% for the combined method while individual datasets enabled at most 82% accuracy. Likewise, Lin et al. reported peak accuracy with fewer variables. In a similar vein, using network-based methodologies, Deng and colleagues further expanded upon the integration of SNP and fMRI data by including DNA methylation data for a cohort of 208 individuals, consisting of 96 patients and 112 healthy controls. The network fusion approach performed better than other methods with various parameter sets (Deng et al., 2017).

### 4.2 Stratification and classification of NDDs

The severity of NDDs varies among patient groups with different ranges of intellectual and verbal disabilities. Individuals with NDDs have a phenotype-genotype correlation restriction due to pleiotropy, insufficient penetrance, and environmental variables (Nussinov et al., 2019b; Zhou et al., 2021). However, advances in computational methods are promising to close the gap between genetics and phenotypic presentation in NDDs with different strategies, such as the usage of causal genes and polygenic risk scores. ML applications were used to discover SNPs in order to develop a predictive classifier that assessed perturbed pathways or biological processes for subgroups in NDDs (Uddin et al., 2019; Song et al., 2022). Common variants that can be protective or pathogenic for ASD were discovered to create an advanced diagnostic classifier for ASD and to verify their classifier. In the DeepAutism study, they selected specific variants among the common variants (more than 1% of the population) from the Simons Simplex Collection (SSC), which consists of 2,600 simplex families, each with one child affected by ASD, unaffected parents, and at least one unaffected sibling (Wang and Avillach, 2021). Convolutional neural network (CNN) models were constructed and trained with Keras and TensorFlow python libraries for artificial neural networks. Both libraries provide developers with the capability to extend functionality across datasets and take more control over the training of machine learning models (Abadi et al., 2016; Wang et al., 2019). They classified cases through common variants, while most studies focused on rare genetic variants. Common variants can be crucial in screening ASD at an early stage (Wang and Avillach, 2021).

Current classification approaches also take polygenic diseases into consideration. For example, individuals with severe and mild ASD were classified using variants with specific functionalities (Talli et al., 2022). Whole exome sequencing (WES) data were assessed with cognitive and language tasks to link genetic variation in translated proteins with specific clinical manifestations. The linear regression polygenic risk score uncovers molecular fingerprints such as genetic profiles and biological processes that are specific to mild and severe ASD. Another study suggested a new approach, netMoST, which constructed allele-specific networks through correlations among SNP-alleles, and identified network modules, and their biological functions in the network (Wei et al., 2023). Following patients’ stratification, it uncovered the associated haplotype biomarkers. To prove their concept, the researchers validated biological subtypes based on neuroimaging. By using SNP, they stratified schizophrenia patients into three subtypes and recognized risk SNP modules.

Overlapping genetic factors in NDDs, cancer, and other developmental disorders suggest shared phenotypic genetic heterogeneity as well as common biological pathways and processes (Grove et al., 2019; Di Giovanni et al., 2023). *De novo* CNVs and SNVs (single nucleotide variants), as well as loss of function mutations, are unquestionably more prevalent in patients than in controls. The main cause of misdiagnosis or missed diagnosis is their complexity. Bioinformatic predictions based on pathogenicity cannot confidently categorize the more frequent missense mutations for disease classification. As a solution, ML algorithms ultimately focus on clusters of specific disease-associated genes. For instance, specific gene clusters in autism spectrum disorder and schizophrenia were demonstrated to have distinguishing genetic features and associated pathways by WES data analysis with the regularized gradient-boosted machines (Sardaar et al., 2020). The algorithms efficiently separated these patients and clustered the genes associated with each disease.

### 4.3 NDD and cancer biomarker/disease-related gene prediction

One of the critical challenges in NDDs is establishing a comprehensive gene list since NDDs have not been studied as thoroughly as cancer. NDD-associated mutations and genomic variants in databases such as OMIM, SFARI, and denovo-db have been growing. Genome-wide studies have mainly focused on *de novo* and transmitted loss of function mutations and assessed genes in the context of brain-specific biology for more specific biomarker prediction. For example, the brain-specific functional relationship network was constructed through Bayesian network integration of various functional genomic data types (Duda et al., 2018). Next, a sophisticated random forest ensemble model ranked candidate genes and identified associated pathways. Another study with a brain-specific functional interaction network constructed a genome-wide probabilistic graph that was composed of genes, pathways, and their functions, integrating numerous genomic experiments (Krishnan et al., 2016). Candidate ASD-associated genes were predicted based on the interaction patterns of known ASD-associated genes. The top predicted genes and their associated CNVs were characterized to find functional modules in the brain.

The impact of an uncommon single mutation is poorly understood in the context of pathogenicity, as well as disease association (Sergouniotis et al., 2016; Landrum et al., 2018). When comparing NDDs and cancer, computational approaches remain inadequate to predict biomarkers due to the scarcity of labeled data and the use of gene level features. To solve this dilemma, SHINE, a pathogenicity prediction tool, recruited protein language models, where protein secondary structure (coil), intrinsically disordered residues, and relative solvent accessibility were transformed into protein statistics (Fan et al., 2023). Also, the tool assessed mutational hotspots on both NDDs and cancer within the test datasets. Learning-based approaches through dimensionality reduction methods such as principal component (Martini et al., 2019; Dugourd et al., 2021), independent component (Liu et al., 2019), and factor analyses (Argelaguet et al., 2018) are able to simplify various statistics. In this way, these reduced features capture potential new information which was not well represented with the complete set of features, and have better performance for a range of prediction tasks than conventional methods based on manually calibrated features (Fan et al., 2023).

A recent study by Pergola et al. (Pergola et al., 2023) aimed to understand how genes associated with schizophrenia work together in specific brain regions at different stages of life. The authors anticipated that during stages of development and aging, these genes would converge into distinct coexpression pathways in specific brain regions. To test this idea, they chose a collection of schizophrenia risk genes based on GWAS (Genome-Wide Association Studies)-significant SNPs and then developed gene modules containing these genes. Then, they analyzed the coexpression of these genes across different age periods in brain regions that are known to be involved in schizophrenia, such as the dorsolateral prefrontal cortex (DLPFC), hippocampus, and caudate nucleus. They discovered that the coexpression patterns of these genes changed over time and were specific to certain brain regions, supporting their hypothesis. Then, they reproduced their analysis using other datasets to confirm their findings and discovered a set of consistent molecular associates of schizophrenia risk genes in these networks. They also investigated if GWAS gene coexpression associations were maintained in induced pluripotent stem cells (iPSCs), which could shed light on mechanisms causing schizophrenia. They revealed 28 genes in the prefrontal cortex that are consistently associated with schizophrenia, 23 of which had not been identified. Interestingly, according to the findings, the hereditary causes of schizophrenia are linked to shifting patterns of gene expression across different parts of the brain and across different ages, which may help explain how the condition manifests itself in individuals and emphasizes expression levels during distinct time windows by distinct cells at specific brain locations.

### 4.4 The role of mutations in the emergence and prognosis of NDDs

Studies implicate germline, *de novo*, and somatic mutations in pathologies, including NDDs (Acuna-Hidalgo et al., 2016; D'Gama and Walsh, 2018; Nishioka et al., 2019; Li et al., 2020; Deb and Bateup, 2021; Kim et al., 2021; Rashed et al., 2022). Several groups have recently explored the impact of different types of mutations, such as missense or truncating mutations, on disease onset and progression; however, mutations associated with NDDs were mostly rare. Analysis of brain tissue from individuals with and without schizophrenia revealed that somatic mutations in brain cells, that is, mutations that occur after fertilization, may play a role with a higher frequency of somatic mutations in certain brain cells (Kim et al., 2021). Although occurring at a low level–affecting only a small proportion of cells in the brain–these mutations were found to affect genes associated with neuronal development and function (Kim et al., 2021). Since NDD mutations are typically weak, the numbers could be higher than those detected.

Comprehensive computational exome sequencing analysis also revealed a number of genes, six of which (*SETD1A*, *CUL1*, *XPO7*, *GRIA3*, *GRIN2A*, and *RB1CC1*) had odds ratios higher than ten, with their rare mutations markedly increasing the risk for schizophrenia (Nakamura and Takata, 2023). Several disease models with high etiological validity have been constructed in light of these discoveries and the earlier identification of CNVs with comparably significant outcomes (Nakamura and Takata, 2023). Mutation burden tests and logistic regression were used to determine whether rare genetic variants contribute to the risk of schizophrenia and whether this risk differed between individuals with and without intellectual disability (Singh et al., 2017). Analysis of rare genetic variants in 6,894 people with schizophrenia, 2,331 people with intellectual disabilities, and 10,963 healthy people discovered that rare genetic variants were more strongly associated with schizophrenia in people with intellectual disabilities than in people without. The mutations were related to brain development, including in genes involved in neuronal migration and synapse formation. Individuals with intellectual disability and schizophrenia were more likely to have rare genetic variants in genes linked to schizophrenia. This suggests that mutations associated with intellectual disability may increase the risk of schizophrenia in people who already have a genetic predisposition, possibly via shared proteins and pathways.

Teng et al. (Teng et al., 2018) explored the genetic overlap and pleiotropy of cognitive function and neuroticism in psychiatric disorders such as schizophrenia, BD, and major depressive disorders by using mutation burden analysis. Genes involved in brain development showed different phenotypic associations, with an increase of disruptive variants in schizophrenia in the *DISC1* gene, resulting in lower cognitive ability at an early age. Similarly, *SMARCC2* (a gene encoding BRG1-associated factor 170, BAF170), with a vital role in corticogenesis and embryogenesis, is the key regulator of the ATP-dependent chromatin remodeling BAF complex. Whole genome sequencing identified 13 heterozygous mutations in *SMARCC2* which are shown to be *de novo* and had given rise to neurodevelopmental delay and growth retardation in 15 individuals (Machol et al., 2019).

Whole-exome sequencing on 13,933 patients with major BD and 14,422 controls identified ultra-rare protein-truncated variants (PTVs) in both BD major subtypes (bipolar I disorder and bipolar II disorder). In genes with significant evolutionary constraints, the study found an excess of ultra-rare PTVs in BD patients (Palmer et al., 2022). A truncating mutation of chromodomain helicase DNA-binding protein 8 (CHD8) represents one of the strongest known risk factors for ASD (Sugathan et al., 2014). The Schizophrenia Exome Sequencing Meta-analysis (SCHEMA) revealed CHD8 binding sites for *DYRK1A*, *CUL3*, *GRIN2B*, *POGZ* (Cotney et al., 2015) and enrichment of ultra-rare PTVs. There are no statistical BD genome-wide association studies (GWASs). AKAP11 (A-kinase anchoring protein 11), a clear risk gene shared with schizophrenia that interacts with GSK3B, the presumed target of lithium, is the main treatment target for BD (Palmer et al., 2022).

Another computational study that examined the full exomes of 24,248 schizophrenia cases and 97,322 controls observed that some ultra-rare coding variations (URVs) confer a substantial risk for schizophrenia, with odds ratios ranging from 3 to 50 and a *p*-value of 2.14 × 10^−6^. The study links 32 genes with a 5% false discovery rate in central nervous system neurons and various biochemical activities to schizophrenia, and links 10 genes with schizophrenia risk. The results support the idea that schizophrenia etiology involves disruption of the glutamatergic system (Singh et al., 2022).

253 genes were observed to be potentially associated with neurodevelopmental diseases with an excess of missense and/or probable gene-disrupting mutations, according to exome sequence data from about 10,000 people with autism spectrum disorder, intellectual disability, and/or developmental delay. The studies utilized gene expression and protein-interaction networks (Coe et al., 2018). To learn more about the potential contribution of *de novo* mutations to the etiology of BD, a trio-based exome sequencing investigation was carried out. 71 *de novo* point mutations and one *de novo* copy-number mutation were discovered in 79 BD probands. There was a notable enrichment of genes resistant to protein-altering variations among genes affected by *de novo* loss-of-function or protein-altering mutations. Brain disorders and schizoaffective illnesses both have a global enrichment of *de novo* mutations, according to a combined analysis including data on schizoaffective disorders. *De novo* protein-altering mutations in BD probands led to noticeably earlier disease onset compared to non-carriers (Kataoka et al., 2016).

## 5 Approaches to validate the NDD-cancer connection hypothesis

We hypothesized that one major cause for the emergence of NDDs and cancer—two diseases (or conditions) with vastly different clinical features—is the mutation load in the same genes. To validate our hypothesis computationally, firstly we identified mutations that are common between NDDs and cancer, as well as the mutations that are preferentially observed in both diseases. Then, we concentrated on PTEN and PI3Kα, two proteins with NDD and cancer mutations that we are familiar with their structural and dynamic properties.

We investigated the domain distributions and the locations in the 3D structure of common mutations and the mutations occurring only in one of the diseases. We observed that cancer mutations accumulate in critical regions of these proteins that fully thwart PTEN’s tumor-suppressive activities while promoting PI3Kα′s oncogenicity (Jang et al., 2021; Jang et al., 2023a). PTEN’s tumor suppressive functions are also partially tallied in the presence of PTEN mutations in NDD samples. As to PI3Kα mutations that are present in NDD patients, we evaluated their oncogenic potential. In line with our thesis, we observed that they appear incapable of promoting tumorigenesis on their own.

This approach could be extended to all genes harboring common mutations and to mutations that are harbored by either NDDs or cancer. Molecular dynamics (MD) simulations of commonly mutated gene products can point to their potential consequences, although conducting the simulations requires a sufficiently long time (Jang et al., 2023a). Taketomi et al. employed MD simulations to reveal a novel mechanism relating a point mutation in the *SPARCL1* gene to the molecular and cellular characteristics associated with ASD (Taketomi et al., 2022). To investigate the importance of microtubule-associated protein 2 (MAP2) phosphorylation in schizophrenia, the authors performed a phosphoproteomic analysis of MAP2 in the primary auditory cortex of schizophrenia and nonpsychiatric control (NPC) patients. Using network analysis, they discovered 18 distinct phosphopeptides and divided them into three modules, each with a unique link to the respective pathology. They also used MD simulations to go further into the most changed phosphorylation location, serine 1782 (pS1782), and discovered that phosphomimetic alteration at this point lowers microtubule interaction (Grubisha et al., 2021). We applied simulations to PTEN to figure out the mechanisms of cancer- and NDD-related mutations and differentiate between them (Jang et al., 2023a), with the aim of *a priori* identifying the mutations. Our premise was that strong oncoprotein mutational variants tend to visit the active, catalytic state more often than NDD variants do. Another metric for evaluating mutation strength is utilizing variant-associated prediction tools, in our case, MutPred2 (Pejaver et al., 2020). Such tools merge experimental data with predictions and classify the mutations as harmful or benign with a pathogenicity score varying from 0 to 1.

Much data has accumulated through single-cell and spatial omics studies of cancer (Lee et al., 2014; Lei et al., 2021; Zeng et al., 2022; Camps et al., 2023), which are lagging for NDDs. Single-cell sequencing technologies have enabled a groundbreaking resolution of omics where bulk-sequencing data fall short. Single-cell based methods can address areas ranging from revealing the tumor, and the tumor microenvironment (TME) heterogeneity (Pan et al., 2020; Tian et al., 2022), to identify subpopulations and cell states (Figure 3). They also offer a venue for a more comprehensive investigation of oncogenic mechanisms (Pan et al., 2020). Single cell spatial transcriptomics can inform cell-to-phenotype over time mapping by capturing the physical tissue structure, which is of special importance for tissues like the brain (Khodosevich and Sellgren, 2023; Piwecka et al., 2023; Vandereyken et al., 2023). These emerging technologies have unrivaled promise for signatures of tumor growth and progression (Pan et al., 2020; Tian et al., 2022). They allow higher-resolution analysis, which could dramatically illuminate molecular processes that fuel malignancies, aiding cancer surveillance systems and treatment approaches (Piwecka et al., 2023; Vandereyken et al., 2023).

These technologies could also be useful in NDDs, improving the understanding of human brain development. This can help in modeling NDDs and more precisely depicting key developmental disorders (Nowakowski et al., 2017; Moffitt et al., 2018; Fan et al., 2020; Kim et al., 2020; Eze et al., 2021; Khodosevich and Sellgren, 2023). For example, a single-cell based analysis by Skene et al. showed that common schizophrenia GWAS variants, and previously identified schizophrenia-associated genes, mapped to certain groups of brain cell types with a much higher frequency than others, hinting at the distinctive roles of different cells (Skene et al., 2018). Maynard et al. combined single-cell and spatial transcriptomics data of the six-layered human dorsolateral prefrontal cortex (DLPFC) and revealed layer-enriched expression signatures. Addition of schizophrenia and ASD-related genes to the analysis uncovered the presence of differential layer-enriched expression indicating clinical importance (Maynard et al., 2021). Replication time of single-cell RNA sequencing (scRNA-seq) data showed that gene clusters linked to cancer and ASD are restrained in late replication (Nassir et al., 2021). International efforts like The Human Cell Atlas Project, which aims to create a comprehensive molecular map of all human cells to aid studies of physiological states, could help in grasping the origin of cellular dysregulation (Regev et al., 2017).

## 6 Comparison of cancer and NDDs is challenging

Due to the heterogeneous nature of cancer and NDDs, it is challenging to draw parallels between two diseases. Exploiting all cancer types and NDD phenotypes implies bigger datasets to learn from; the heterogeneity increases the complexity. Narrowing the comparison to a single phenotype from both diseases may enable discoveries that would have been missed otherwise. This is especially the case for NDDs whose phenotypes overlap, resulting in different phenotypes observed by different researchers. While data availability is crucial in determining phenotypes, especially for NDDs, recent epidemiological cohorts provided convenient starting points. In a cohort study of 8,438 patients with autism, Chiang et al. observed an increased risk of genitourinary and ovarian cancers (Chiang et al., 2015). Another cohort study reported a higher risk of breast cancer in BD patients and their healthy siblings in comparison to the control (Chen et al., 2022), again in line with our thesis of the possible connection between cancer and NDDs. To uncover the underlying mechanisms, the phenotypes to be studied can be selected based on already reported connections, such as thyroid cancer and autism, or breast cancer (Figure 1A) and BD.

## 7 Conclusion

Computations can analyze the rapidly increasing volume of data, identify trends, and derive correlations at a scale that experiments are unable to do. By discovering causal relationships, they can also identify the most probable events, processes, states, and objects. In our case, such discoveries are extremely consequential as they can identify the likelihood of NDDs early in life *even prior to emergence of the debilitating phenotype*. No less important, they can project the likelihood of cancer, assisting with early pharmacology. Cancer onset is associated with abnormal cell proliferation (Brunner and Finley, 2023; de Visser and Joyce, 2023), while NDDs are mostly related to dysregulated differentiation (Khodosevich and Sellgren, 2023). In terms of cell cycle signaling (Demeter et al., 2022), they differ in signaling strength and duration (Yavuz et al., 2023a). Under physiologic conditions, mitogen-promoted strong signaling bursts over short duration are associated with cell proliferation. Weaker, extended mitogen-promoted signaling is associated with cell differentiation. When disease-related, due to activating mutations or overexpression, signaling is constitutive, thus always extended. When strong, the outcome is likely to be proliferation in cancer. The paramount factor is the population of proteins in their active states. Both strong, mitogen-promoted bursts, and mutation-elicited, lead to a larger population in the active state (Nussinov et al., 2022a). Higher expression level due to, e.g., gene duplication, or dysregulation, lead to the same outcome.

Cancer and NDDs are not the only diseases associated with cell cycle and signaling strength. Neurodegenerative diseases in old age, cardiovascular disease (Wiman and Zhivotovsky, 2017), vascular proliferative disorders (Sriram and Patterson, 2001), diseases with prolonged mitosis (Levine and Holland, 2018), possibly the outcome of low translation rates, and more, can all be related to the cell cycle.

Here, we discussed computational approaches, including AI, ML, and MD simulations, as well as others, such as networks construction, and their findings. Computational observations based on ‘large enough’, high quality data, can be powerful. We took up the linkage between NDDs and cancer, and emerging strategies for pursuing them. Our comprehensive searches of the literature uncovered an abundance of experimental and clinical studies. However, unfortunately, we failed to identify computational approaches, especially at the detailed molecular level addressing questions such as those that we raised above, leading us to describe the ones that we have adopted and possible extensions. We hope that these open the door to future studies of this emerging innovative and enthralling computational discipline.

Taken together, our broad review underscores the multi-scale aspect of this type of reasoning, rather than focus on a specific organ system or disease, with the examples that are used demonstrating what new understanding can be gained.
